# Characterization of the far-red fluorescent probe MitoView 633 for dynamic mitochondrial membrane potential measurement

**DOI:** 10.3389/fphys.2023.1257739

**Published:** 2023-10-23

**Authors:** Patrick Ernst, Seulhee Kim, Zengqiao Yang, Xiaoguang Margaret Liu, Lufang Zhou

**Affiliations:** ^1^ Department of Medicine, University of Alabama at Birmingham, Birmingham, AL, United States; ^2^ Department of Biomedical Engineering, The Ohio State University, Columbus, OH, United States; ^3^ Department of Surgery, The Ohio State University, Columbus, OH, United States; ^4^ Department of Chemical and Biomolecular Engineering, The Ohio State University, Columbus, OH, United States

**Keywords:** molecular probe, live cell imaging, mitochondria, energetics, confocal

## Abstract

**Introduction:** MitoView 633, a far-red fluorescent dye, exhibits the ability to accumulate within mitochondria in a membrane potential-dependent manner, as described by the Nernst equation. This characteristic renders it a promising candidate for bioenergetics studies, particularly as a robust indicator of mitochondrial membrane potential (DY_m_). Despite its great potential, its utility in live cell imaging has not been well characterized.

**Methods:** This study seeks to characterize the spectral properties of MitoView 633 in live cells and evaluate its mitochondrial staining, resistance to photobleaching, and dynamics during DYm depolarization. The co-staining and imaging of MitoView 633 with other fluorophores such as MitoSOX Red and Fluo-4 AM were also examined in cardiomyocytes using confocal microscopy.

**Results and Discussion:** Spectrum analysis showed that MitoView 633 emission could be detected at 660 ± 50 nm, and exhibited superior thermal stability compared to tetramethylrhodamine methyl ester (TMRM), a commonly used DY_m_ indicator, which emits at 605 ± 25 nm. Confocal imaging unequivocally illustrated MitoView 633’s specific localization within the mitochondrial matrix, corroborated by its colocalization with MitoTracker Green, a well-established mitochondrial marker. Furthermore, our investigation revealed that MitoView 633 exhibited minimal photobleaching at the recommended *in vitro* concentrations. Additionally, the dynamics of MitoView 633 fluoresce during carbonyl cyanide-p-trifluoromethoxyphenylhydrazone (FCCP, a mitochondrial uncoupler)-induced DY_m_ depolarization mirrored that of TMRM. Importantly, MitoView 633 demonstrated compatibility with co-staining alongside MitoSOX Red and Fluo-4 AM, enabling concurrent monitoring of DY_m_, mitochondrial ROS, and cytosolic Ca^2+^ in intact cells.

**Conclusion:** These findings collectively underscore MitoView 633 as a superb molecular probe for the singular or combined assessment of DY_m_ and other indicators in live cell imaging applications.

## Introduction

Mitochondria assume multifaceted roles vital for maintaining cellular function and overall health. These roles encompass the augmentation of energy production in response to increased workload (Zhou et al., 2007), buffering cytosolic Ca^2+^ under physiological conditions (Contreras et al., 2010; Wei et al., 2011), and the intricate regulation of apoptosis, a form of programmed cell demise (Honda et al., 2005). Any impairment in mitochondrial function precipitates disruptions in cellular metabolism and an upsurge in reactive oxygen species (ROS), a recognized catalyst for cellular and organ dysfunction (Uttara et al., 2009; Wallace, 2012). The maintenance of a consistent electrical potential across the inner mitochondrial membrane, known as the mitochondrial membrane potential (ΔΨ_m_), stands as a linchpin for normal mitochondrial function. Hence, substantial alterations in ΔΨ_m_ could serve as a valuable biophysical indicator of overall cellular wellbeing. For instance, studies have shown that when exposed to significant oxidative stress, metabolic processes become susceptible to perturbations, leading to mitochondrial oscillations within cardiomyocytes (Zhou et al., 2010; Zhou et al., 2011; Zhou and O'Rourke, 2012).

Presently, the measurement of plasma membrane potential (V_m_) is conventionally conducted through methodologies like patch clamping (Segev et al., 2016). However, this technique cannot easily be translated to mitochondrial ΔΨ_m_ measurement, as it necessitates the extraction of mitochondria from viable cells and their suspension in a medium designed to mimic the intracellular environment but inevitably distinct from it (Bertholet et al., 2017; Sorgato et al., 1987; Xu et al., 2002). Moreover, this isolation process carries the potential for substantial disruption to mitochondrial membrane integrity, thereby complicating the interpretation of observed outcomes. Consequently, the evaluation of mitochondrial ΔΨ_m_ within living cells predominantly hinges on the application of fluorescent probes. These probes, typically composed of lipophilic cationic compounds, diffuse through the plasma membrane and undergo iontophoresis towards the negative potential ΔΨ_m_, subsequently accumulating within the mitochondrial matrix space following the Nernst equation:

$$V=-\frac{RT}{ZF}\ln\left(\frac{C_{i}}{C_{o}}\right)$$

Where R is the gas constant, T is the temperature in Kelvin, Z is the charge of the ionic species in question, F is Faraday’s constant, and C_i_ and C_o_ are the dye concentrations inside and outside of the mitochondrial matrix, respectively. As such, more negative (i.e., more polarized) ΔΨ_m_ will accumulate more dye, and *vice versa*.

A group of fluorescent probes commonly employed to assess mitochondrial ΔΨ_m_ includes derivatives of the fluorone dye rhodamine, namely, rhodamine 123 (R123), tetramethylrhodamine methyl ester (TMRM), and tetramethylrhodamine ethyl ester (TMRE), all of which demonstrate specific accumulation within mitochondria. However, a potential limitation of these dyes becomes apparent at relatively high concentrations (e.g., >50 nmol/L), with TMRE showing the most pronounced suppressive effect on mitochondrial respiration, followed by R123, as noted in previous studies (Scaduto and Grotyohann, 1999; Perry et al., 2011). Two other frequently utilized probes for assessing ΔΨ_m_ are 5,5′,6,6′-tetrachloro-1,1′,3,3′-tetraethylbenzimidazolyl carbocyanine iodide (JC-1) (Reers et al., 1991) and DiOC_6_ (BERNAS et al., 2004; Korchak et al., 1982). JC-1 can form monomers or aggregates, which exhibit fluorescent emission at 529 nm and 590 nm, respectively. It serves as a ratiometric dye, as the 590/529 emission ratio diminishes significantly during substantial depolarization when it shifts back to its monomeric state, as described by Garner and Thomas (Garner and Thomas, 1999). Nonetheless, JC-1 has its limitations, as its aggregated form has been reported to respond to factors beyond mitochondrial ΔΨ_m_, such as H_2_O_2_ and surface area-to-volume ratios, potentially leading to misinterpretation of potential differences (Perry et al., 2011). DiOC_6_ (BERNAS et al., 2004) is a cyanine dye most commonly used for flow cytometric analysis (Shapiro, 2000; Perry et al., 2011). However, it is not well-suited for other applications due to its requirement for extremely low concentrations (<1 nmol/L), as higher concentrations can impede respiration.

While TMRM proves to be an effective probe for assessing mitochondrial ΔΨ_m_, its limitation lies in its red fluorescent spectrum, which constrains its simultaneous use with other red fluorescent probes to observe related physiological processes. One notable example is the red fluorescent superoxide indicator MitoSOX Red, a mitochondria-targeted dye widely employed for live cell detection of ROS levels (Hanninen et al., 2010; Andersson et al., 2011; Polster et al., 2014). Given the well-established connection between perturbations in mitochondrial ΔΨ_m_ and the dynamics of ROS, the capacity to concurrently monitor their changes holds significant promise for gaining deeper insights into various pathological cellular conditions. MitoView, a family of fluorescent mitochondria dyes manufactured by Biotium (https://biotium.com), has been recently growing in popularity. Like TMRM and other rhodamine derivatives, MitoView dyes permeate cell membranes and rapidly accumulate within the mitochondrial matrix, with fluorescence intensity increasing in proportion to their concentration. This family comprises a total of five MitoView dyes, each featuring distinct fluorescent spectra, including blue, green, two far-red, and one near-infrared variant. While most of these dyes exhibit minimal voltage dependence, one of the far-red variants, MitoView 633, does display such characteristics.

While MitoView 633 is commonly utilized for mitochondrial localization (Ishii and Rohrer, 2017; Li et al., 2017; Min et al., 2018), recent applications have extended to ΔΨ_m_ measurement (Maioral et al., 2017; Ernst et al., 2019; Sciuto et al., 2019; Kim et al., 2020; Ernst et al., 2021). Thanks to its far-red spectrum, it stands as an excellent candidate for fluorescently monitoring mitochondrial ΔΨ_m_, particularly when used alongside other red-spectrum fluorescent indicators like MitoSOX Red. In this study, we aim to thoroughly characterize and assess the potential of MitoView 633 as a robust mitochondrial ΔΨ_m_ indicator, and compare its performance with TMRM, a conventional, widely used ΔΨ_m_ dye in live cell imaging.

## Methods

All experimental protocols involving animals were approved by the University of Alabama at Birmingham institutional animal care and use committee and adhered to the National Institutes of Health’s guide for the care and use of laboratory animals (NIH Publication No. 85-23, revised 2011).

### Cell culture

H9C2 cells were cultured in Dulbecco’s Modified Eagle Medium (DMEM) (Gibco Laboratories, Gaithersburg, MD) supplemented with 10% (v/v) Fetal Bovine Serum (FBS) (Gibco Laboratories), 2 mmol/L L-Glutamine (Gibco Laboratories), and 1X Penicillin-Streptomycin (P/S) (100 U/mL of penicillin and 100 μg/mL of streptomycin) Solution (EMD Millipore, Burlington, MA). AC16 cells were cultured DMEM/F12 (Sigma, St Louis, MO) containing 2 mmol/L L-Glutamine, 12.5% FBS, and 1X P/S Solution. Cells were maintained in 5% CO_2_ at 37°C with a confluence between 10% and 80%. Induced pluripotent stem cell-derived cardiomyocytes (iPSC-CMs) were reprogrammed from human cardiac fibroblasts, following the procedure outlined in Zhao et al. (Zha et al., 2018). They were cultured on Matrigel Membrane Matrix in mTeSR™ medium until reaching 75% confluence. Cardiomyocyte differentiation was achieved using a small molecule-based protocol, as previously described (Ernst et al., 2021). Beating iPSC-CMs typically emerged 9–12 days after the initiation of differentiation. To purify the iPSC-CM cultures and eliminate non-cardiomyocytes, a glucose-free medium containing lactate was employed. Following purification, iPSC-CMs were maintained in RPMI 1640 supplemented with B27 Supplement and 1X P/S. All iPSC-CMs utilized in experiments were within 3 weeks of differentiation.

### Adult cardiomyocyte isolation

Mouse cardiomyocytes were isolated from 8-week-old C57BL/6 mice *via* Langendorff perfusion, as previously described (Goh et al., 2019; Kim et al., 2020). Briefly, following thoracotomy, hearts were quickly excised, mounted on a Langendorff apparatus, and perfused with Tyrode’s solution containing (in mmol/L) 113 NaCl, 4.7 KCl, 0.6 KH_2_PO_4_, 0.6 Na_2_HPO_4_, 1.2 MgSO_4_, 10 Na-HEPES, 12 NaHCO_3_, 10 KHCO_3_, 0.032 phenol red, 30 taurine, 10 2,3-Butanedione monoxime, and 5.5 glucose at 37°C for 3–5 min. Thereafter, the heart was digested with collagenase II (Worthington Biochemicals, Lakewood, NJ) for 10–12 min. The digested heart was pulled apart with forceps into tiny pieces and then piped in digestion solution several times to further break up cell clusters. The cell solution was filtered through a 100 μm cell strainer to remove any undigested tissue. Next, cardiomyocytes were introduced to grade-elevating concentrations of CaCl_2_ (in μmol/L: 12.5, 100, 400, and 900) to establish Ca^2+^ tolerance. Finally, isolated cells were stored in a high K^+^ solution (in mmol/L: 120 Glutamate, 25 KCl, 1 MgCl_2_, 10 HEPES, 1 EGTA, and pH 7.5 with KOH) temporarily before fluorescent dye loading and experiments.

### Spectral measurement

Determination of MitoView 633 excitation and emission spectra was performed using a Spectramax i3x microplate reader (Molecular Devices, San Jose, CA). Cells were loaded with MitoView 633 of a desired concentration. Fifteen minutes later, the dye-containing medium was replaced with a colorless culture medium titrated to the indicated pH before spectral measurement. The temperature was adjusted using Spectramax’s internal temperature control. Excitation spectra were determined by measuring 720 nm emission using excitation light from 500–700 nm in 10 or 2 nm increments. Emission spectra were determined by measuring emission from 600–800 nm in 10 or 2 nm increments using excitation light at 580 nm. All spectra (absorption and emission) were normalized to their maximum values.

### ATP content measurement

The Cellular ATP content was quantified employing the CellTiter-Glo Luminescent Viability Assay (Promega, Madison, WI). In brief, an equivalent number of cells were seeded into 96-well plates on Day 0. On Day 1, cells were exposed to Mitoview 633 at varying concentrations (ranging from 0 to 200 nmol/L) for a 30-min incubation period. Following incubation in darkness, cells were rinsed and resuspended in 100 μL of phenol red-free DMEM. Simultaneously, an ATP standard curve was generated using ATP disodium salt (Sigma Cat. # A7699) within the same plate on which stained cells were seeded. Subsequently, 100 μL of CellTiter-Glo reagent was introduced into each well, followed by a brief 2–3-min agitation of cellular contents on an orbital shaker. The plate was then allowed to incubate for 10 min to stabilize the luminescent signal, after which luminescence was measured using a SpectraMax microplate reader.

### Confocal imaging acquisition and data analysis

Cells (adult mouse cardiomyocytes or iPSC-CMs) were loaded with MitoView 633 (25 nmol/L for 15 min at 37°C), MitoTracker Green (250 nM for 30 min at 37°C), or TMRM (20 nmol/L for 15 min at 37°C). The glass-bottom dish containing dye-loaded cells was equilibrated at 37°C (or specified temperature) with unrestricted access to atmospheric oxygen on the stage of an Olympus FV1000 confocal microscope (Olympus, Center Valley, PA). The 488 nm argon laser, 543 nm HeNe laser, and 635 nm LD laser lines were used to image MitoTracker Green, TMRM, and MitoView 633, respectively. The emission filters used to collect images were 505–605 nm, 560–620 nm, and 655–755 nm, respectively. Colocalization of Mitotracker Green and MitoView 633 was analyzed using the previously described method (Kim et al., 2020).

To access the performance of MitoView 633 in measuring stress-induced ΔΨ_m_ depolarization, cells loaded with MitoView 633 alone or in conjunction with TMRM were deposited onto a cover glass in a microincubation system (Harvard Apparatus, Cambridge, MA). The chamber was held at 37°C and perfused with Tyrode’s solution containing (in mmol/L): 137 NaCl, 2.7 KCl, 1 MgCl_2_, 20 HEPES, 4.5 glucose, 1.8 CaCl_2_, pH 7.4, at a flow rate of 1 mL/minute. Mitochondrial depolarization was induced by introducing FCCP into the perfusate at a final concentration of either 1 or 10 μmol/L. The 543 nm HeNe laser and 635 nm LD laser lines were used to image TMRM and MitoView 633, respectively.

To simultaneously measure ΔΨ_m_, ROS levels, and cytosolic Ca^2+^, isolated cardiomyocytes were loaded with MitoSOX Red (2 μmol/L), MitoView 633 (25 nmol/L), and Fluo4-AM (5 μmol/L for 30 min, followed by a 30-min wash out to allow for de-esterification). The laser flash method described previously (Aon et al., 2003; Zhou et al., 2011; Goh et al., 2016) was adopted to elicit local oxidative stress and mitochondrial depolarization. In brief, a high-intensity laser flash (453 nm, ∼25 mW, 100 ms duration) was applied to a small (∼100 μm^3^) region within the cell volume. After photoactivation, Fluo4-AM, MitoSOX Red, and MitoView 633 were concurrently imaged using the 488 nm argon laser, 543 nm HeNe laser, and 635 nm LD laser, respectively with x60 oil immersion lens (numerical aperture = 1.4). For fast time resolution of Ca^2+^ sparks, the linescan mode of the confocal microscope was used. The linescan rate was 2.0 ms per line; and 512 × 512 pixel xy images were acquired every 3.7 s, as previously described (Zhou et al., 2011).

Images were processed offline using ImageJ software (Wayne Rasband, NIH) or Matlab (The MathWorks, Inc., Natick, MA).

### Statistical analysis

Comparisons were performed using paired or unpaired 2-tailed Student’s t-test. Data were considered significantly different at *p* < 0.05. Results are presented as mean ± SEM.

## Results and discussion

### Characterization of MitoView 633 spectra

**FIGURE 1:**
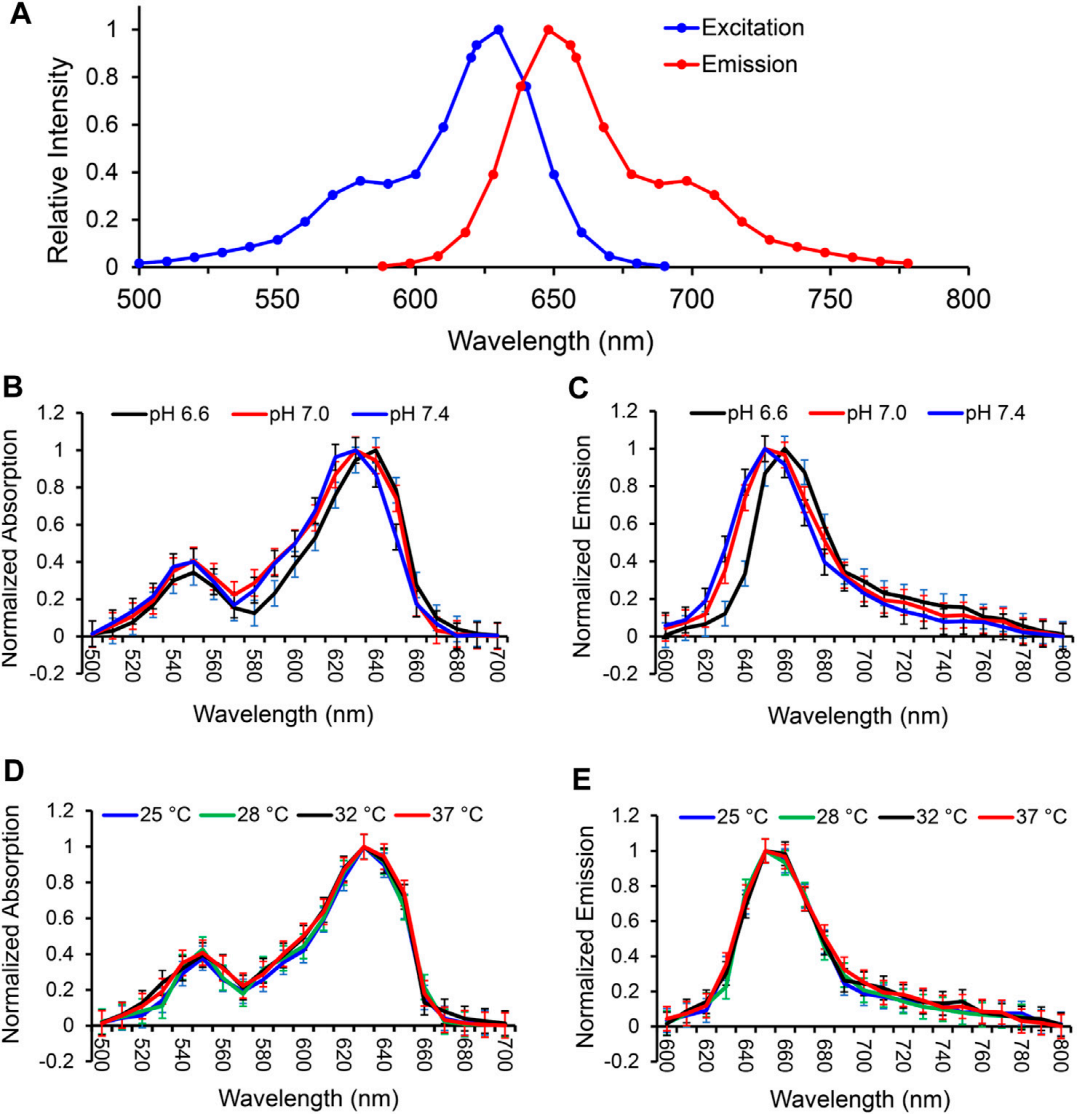
Characterization of MitoView 633 fluorescent spectra in H9C2 cells. **(A)** The excitation and emission spectra of MitoView 633 determined by a Spectramax i3x microplate reader. **(B, C)**: The excitation **(B)** and emission **(C)** spectra of MitoView 633 under varying extracellular pH values (6.6–7.4). **(D, E)**: The excitation **(D)** and emission **(E)** spectra of MitoView 633 under varying temperatures (25°C–37°C). Absorption and emission were normalized to their maximal values.

We first analyzed the spectrum of MitoView 633 in H9C2 cells using the Spectramax i3x microplate reader. The results showed that MitoView 633 excitation and emission spectrum peaked at 622 nm and 648 nm, with the full width at half maximum (FWHM) of 20 nm and 25 nm, respectively, at 37°C and pH 7.4 in colorless culture medium (Figure 1A). These data were consistent with the specifications provided by the manufacturer.

**FIGURE 2:**
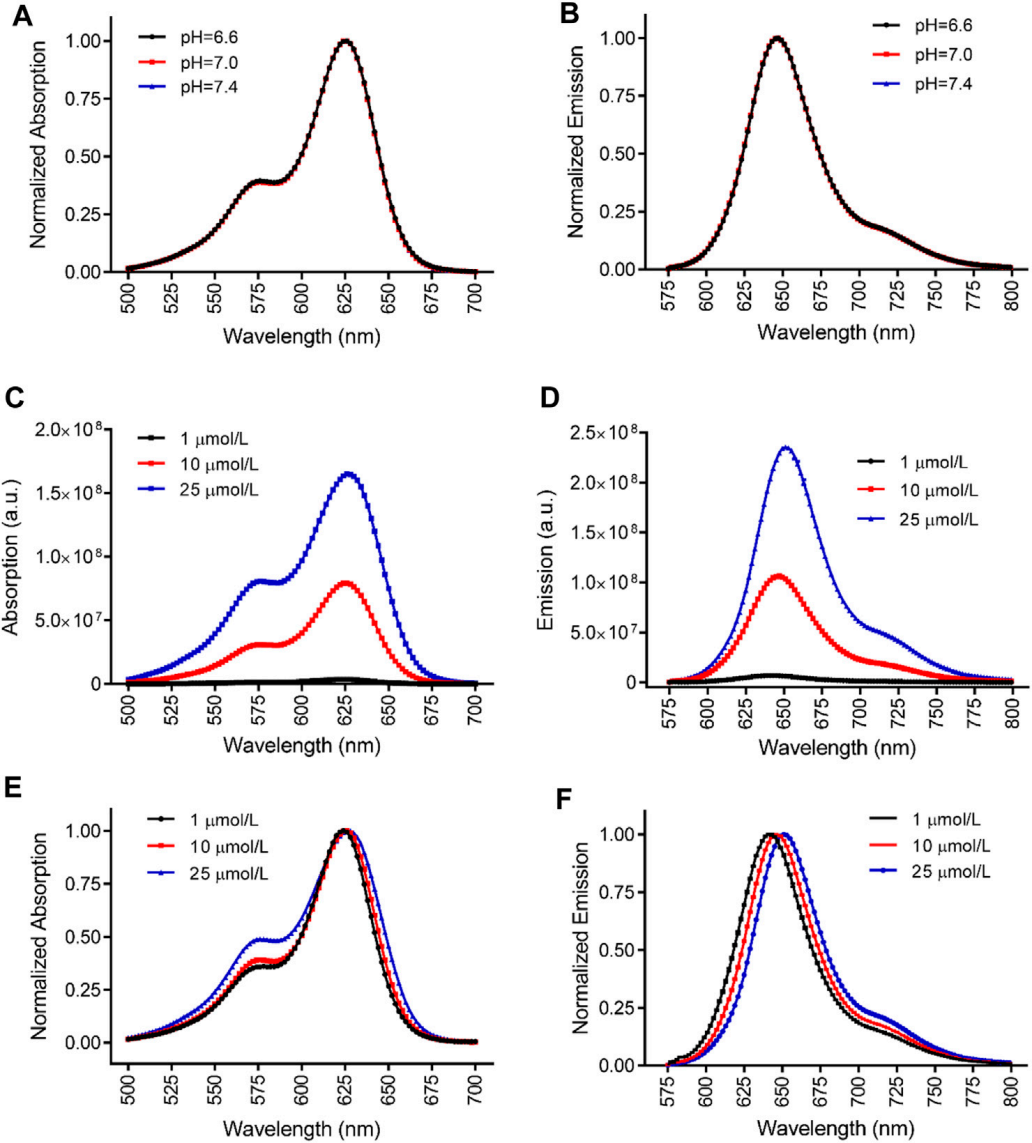
Characterization of MitoView 633 fluorescent spectra in cell-free medium. **(A, B)**: Effect of medium pH on the excitation **(A)** and emission **(B)** spectra of MitoView 633. **(C, D)**: Effect of dye concentration on the absorption **(C)** and emission **(D)** of MitoView 633. **(E, F)**: Effect of dye concentration on the absorption **(E)** and emission **(F)** normalized to the maximal values. *n* = 4 per group.

To investigate the influence of extracellular pH on the fluorescent spectra of MitoView 633, we conducted measurements in H9C2 cells at 37°C, using a colorless culture medium adjusted to pH levels of 6.6, 7.0, or 7.4. At a neutral pH of 7.4, both spectra exhibited a minor blue shift in the peak wavelength, approximately 5 nm, along with a slight reduction in bandwidth, resulting in a 5 nm narrower full width at half maximum (FWHM) when compared to the neutral pH of 7.0 (Figures 1B, C; Table 1). Conversely, at a lower pH of 6.6, we observed an approximate 5 nm red shift in the peak wavelength compared to the neutral pH. The bandwidth at pH 6.6 showed a more pronounced narrowing, resulting in an FWHM 10 nm narrower than that at neutral pH (Table 1). It's important to note that changes in extracellular pH may not accurately reflect intracellular pH, especially in the vicinity of mitochondria or within the mitochondrial matrix. We proceeded to evaluate the impact of pH on MitoView 633 spectra in a cell-free medium. As depicted in Figures 2A, B, alterations in pH had minimal influence on both the excitation and emission spectra of MitoView 633, unlike the observations in cells. Given that the loading of MitoView 633 into the mitochondrial matrix relies on the potential gradient across the membrane, we further examined the MitoView 633 spectra under varying dye concentrations in the cell-free medium. As anticipated, increasing concentrations of MitoView 633 resulted in more pronounced fluorescence signals (Figures 2C, D). Interestingly, it became apparent that dye concentration also exerted an impact on the spectra. With increasing concentration from 1 μmol/L to 25 μmlo/L, the peak excitation wavelength shifted slightly from 624 nm to >626 nm, while the peak emission wavelength shifted significantly from 641 nm to 651 nm (Figures 2E, F). The observed red shift in spectra induced by elevated dye concentration mirrored that caused by reduced pH, suggesting a connection between extracellular pH (or plasma membrane potential) and the accumulation of cationic MitoView 633 within mitochondria.

**TABLE 1:** MitoView 633 spectra FWHM values under varying pH conditions. *n* = 6–8/group.

	6.6	7	7.4
Excitation (nm)	56.48 ± 1.72	55.67 ± 1.55	51.5 ± 2.60
Emission (nm)	34.67 ± 1.45	45.83 ± 2.30	45.33 ± 2.05

Temperature dependence was measured in H9C2 cells at neutral pH using Spectramax’s heating capabilities to adjust the culture temperature from 25°C up to 37°C. Neither excitation (Figure 1D) nor emission (Figure 1E) spectra exhibited a significant red- or blue shift across the entire range measured. A small difference in bandwidth was observed between the two most extreme temperatures, amounting to an about 5 nm narrower FWHM at 25°C as compared to 37°C (Table 2). These data indicate MitoView 633’s fluorescent spectral stability across the physiologically relevant temperature and pH ranges. MitoView 633’s spectra stability is a very useful characteristic; it is highly likely that local intracellular variances in either property could not reach such a magnitude without first causing significant off-target physiological effects.

**TABLE 2:** MitoView 633 spectra FWHM values under varying temperature (^o^C) conditions. *n* = 6–8/group.

	25	28	32	37
Excitation (nm)	47.89 ± 1.14	49.62 ± 1.68	52.57 ± 1.45	54.29 ± 2.60
Emission (nm)	45.47 ± 2.08	43.38 ± 1.10	44.25 ± 1.91	45.45 ± 1.51

### Characterization of MitoView 633 mitochondrial staining

**FIGURE 3:**
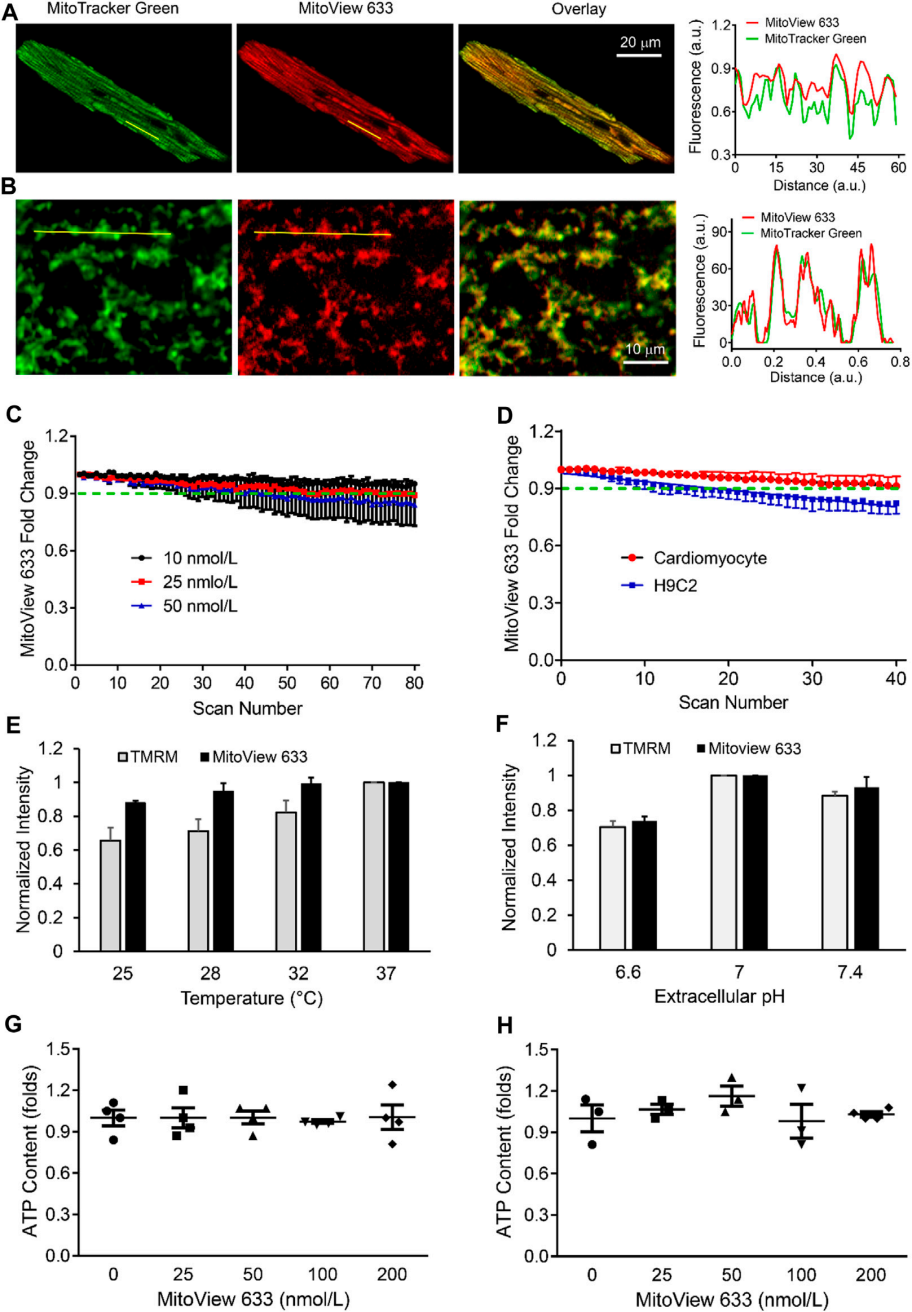
Characterization of MitoView 633 staining and fluorescence stability. **(A, B)**: Representative confocal imaging showing colocalization of MitoView 633 (Ex/Em: 635 nm/664 nm) with MitoTracker Green (Ex/Em: 488 nm/516 nm) in adult cardiomyocytes **(A)** and iPSC-CMs **(B)**. The line scanning imaging revealed overlapping between the peaks of these two fluorophores, confirming MitoView 633’s mitochondrial-specific staining. **(C)** MitoView 633 photobleaching measured at different concentrations. **(D)** Comparison of MitoView 633 photobleaching in adult cardiomyocytes and H9C2 cells. **(E, F)**: Comparison of the thermo **(E)** and pH **(F)** fluorescence stability between MitoView 633 and TMRM. Each group includes 4-6 cells from 4 mice/cell cultures. **(G, H)**: Effect of MitoView 633 loading concentration on cellular ATP content in adult cardiomyocytes **(G)** and AC16 cells **(H)**. *n* = 4 per group.

We next examined the subcellular staining and distribution of MitoView 633 across various cell types. Confocal imaging unveiled that MitoView 633 consistently displayed specific staining within the mitochondria of adult cardiomyocytes. This was confirmed by the colocalization observed with MitoTracker Green, a well-established mitochondrial marker (Figure 3A). To enhance clarity, simultaneous line scan imaging was performed, demonstrating a nearly perfect overlap of the MitoView 633 (red line) and MitoTracker Green (green line) fluorophore peaks (Pearson correlation coefficient, PCC = 0.93 ± 0.04), providing unequivocal evidence of MitoView 633’s mitochondrial localization. The mitochondrial-specific staining of MitoView 633 was also consistently observed in all other tested cell types, including hiPSC-CMs (Figure 3B, PCC = 0.97 ± 0.18), as well as myoblast cells H9C2, triple-negative breast cancer cells, and neurons (data not shown). These findings collectively indicate that MitoView 633 possesses the versatility to serve as a universal far-red spectrum mitochondrial indicator.

Photobleaching represents a phenomenon where a fluorophore experiences a permanent loss of its ability to fluoresce due to photon-induced chemical damage and covalent modifications (BERNAS et al., 2004). This occurrence leads to a reduction in signal-to-noise ratio and image resolution, thereby limiting the utility of repeated scanning, often necessary in life science research. To investigate the potential photobleaching effect of MitoView 633, cardiomyocytes were loaded with various concentrations of MitoView 633 and subjected to repeated XY scanning without external stress. Confocal imaging results revealed that at a concentration of 10 nmol/L, the fluorescence intensity of MitoView 633 remained relatively stable throughout the entire scanning period (80 scans, approximately 250 s) (Figure 3C). The slope coefficient of −8.17 ± 0.37 × 10^−5^ indicated no significant change. At a concentration of 25 nmol/L, the fluorescence of MitoView 633 showed minimal variation (<2%) during the initial 10 scans but experienced a decrease of approximately 9% after 80 repeated scans (slope coefficient: −1.32 ± 0.42 × 10^−4^). At 50 nmol/L, the photobleaching effect became more noticeable, resulting in an approximately 15% reduction in fluorescent intensity by the end of repeated scans (slope coefficient: −1.96 ± 0.45 × 10^−3^). These observations emphasize the need for careful consideration of MitoView 633 concentration to mitigate significant photobleaching. It's noteworthy that the extent of photobleaching appears to be influenced by cell type. As illustrated in Figure 3D, the fluorescence of MitoView 633 in H9C2 cells decreased by nearly 18% after 40 scans (slope coefficient: −4.42 ± 0.23 × 10^−3^), whereas in cardiomyocytes, the reduction was less than 9% (slope coefficient: −2.33 ± 0.16 × 10^−3^). Consequently, thorough characterization of this fluorescent probe is crucial when applying it to new cell types.

We next examined the effect of temperature or pH on MitoView 633 fluorescence and compared it with TMRM. A microincubation system was used to control the temperature of cardiomyocytes loaded with MitoView 633 or TMRM. MitoView 633 and TMRM were recorded with confocal microscopy using the same imaging conditions as the temperature was increased incrementally from room temperature (25°C) up to 37°C, allowing time for the temperature to equilibrate across the incubation system. Whereas TMRM fluorescent intensity increased nearly linearly as temperature elevated, MitoView 633 fluorescence showed no significant change when temperature increased from 28°C to 37°C, and only ∼ 10% when increased from 25°C to 37°C (Figure 3E). This effect was approximately one-third of that observed with TMRM. The temperature changes similarly affected the peak intensity of these two dyes in cell free solution. Both the peak excitation and emission intensities of TMRM increased ∼6.7 folds when the temperature was elevated from 25°C to 37°C (Table 3). On the contrary, the peak intensities of MitoView 633 only changed about 4% with the same temperature fluctuations. This enhanced stability of MitoView 633 across the physiologically relevant temperature range could facilitate more sensitive measurements of mitochondrial ΔΨ_m_ at lower temperatures and potentially minimize confounding factors in scenarios where significant temperature fluctuations are encountered during recording. To assess sensitivity to pH change, cells loaded with either TMRM or MitoView 633 were immersed in Tyrode’s solution adjusted to a neutral pH of 7.4, followed by imaging with confocal microscopy. Subsequently, the chamber solution was switched to one with progressively lower (6.6) or higher (7.4) pH levels, allowing adequate time for cellular equilibration to the new pH before imaging. The data indicated that changes in pH exerted similar effects on TMRM and MitoView 633 fluorescence: the fluorescence intensity of both dyes decreased when pH was shifted away from neutrality, either towards acidity or alkalinity (Figure 3F). These observations underscore the importance of exercising caution when measuring ΔΨ_m_ with either MitoView 633 or TMRM under conditions where pH alterations may occur.

**TABLE 3:** Effect of temperature (^o^C) on the excitation and emission peak intensity of TMRM in cell free solution. *n* = 4-5/group.

	25	32	37
Excitation (a.u.)	3.35 ± 0.29 × 10^7^	9.7 ± 0.64 × 10^7^	2.24 ± 0.64 × 10^8^
Emission (a.u.)	3.55 ± 0.3 × 10^7^	9.94 ± 0.71 × 10^7^	2.39 ± 0.78 × 10^8^

We next investigated the potential impact of MitoView 633 on cellular bioenergetics. We exposed isolated adult cardiomyocytes to varying concentrations (ranging from 0 to 200 nmol/L) of MitoView 633 and subsequently measured cellular ATP content using the Cell Titer-Glo Luminescent Viability Assay. The results, as depicted in Figure 3G, revealed no significant difference in ATP levels among the tested groups, even at the higher concentration of 200 nmol/L. These findings underscore the notion that MitoView 633 staining exerts minimal influence on the cellular respiratory chain and overall energetics. Furthermore, we extended our investigation to AC16 cells, a proliferating human cardiomyocyte cell line derived from the fusion of primary cells from adult human ventricular heart tissues (Davidson et al., 2005). As an immortalized cardiomyocyte cell line, AC16 cells are more glycolytic, and the energy requirements and other physiological responses (e.g., relative stress tolerance) are different from adult cardiomyocytes that have preserved myofibrils, t-tubules, and gap junctions. Hence, we also assessed the effects of MitoView 633 staining on AC16 cells. In line with our observations in adult cardiomyocytes, the loading of MitoView 633 did not induce significant alterations in intracellular ATP concentrations within AC16 cells (Figure 3H). Altogether, our data indicate that MitoView 633 can be effectively employed as an indicator for ΔΨ_m_ measurement in live cell imaging without evident effect on overall cellular energetics. It should be noted that previous studies revealing that TMRM or TMRE moderately inhibits mitochondrial respiration were performed with isolated mitochondria; in intact cells, we found that TMRM loading, at a concentration below 100 nmol/L, had a very small effect on cellular ATP (less than 4%).

### Measurement of stress-induced mitochondrial depolarization

**FIGURE 4:**
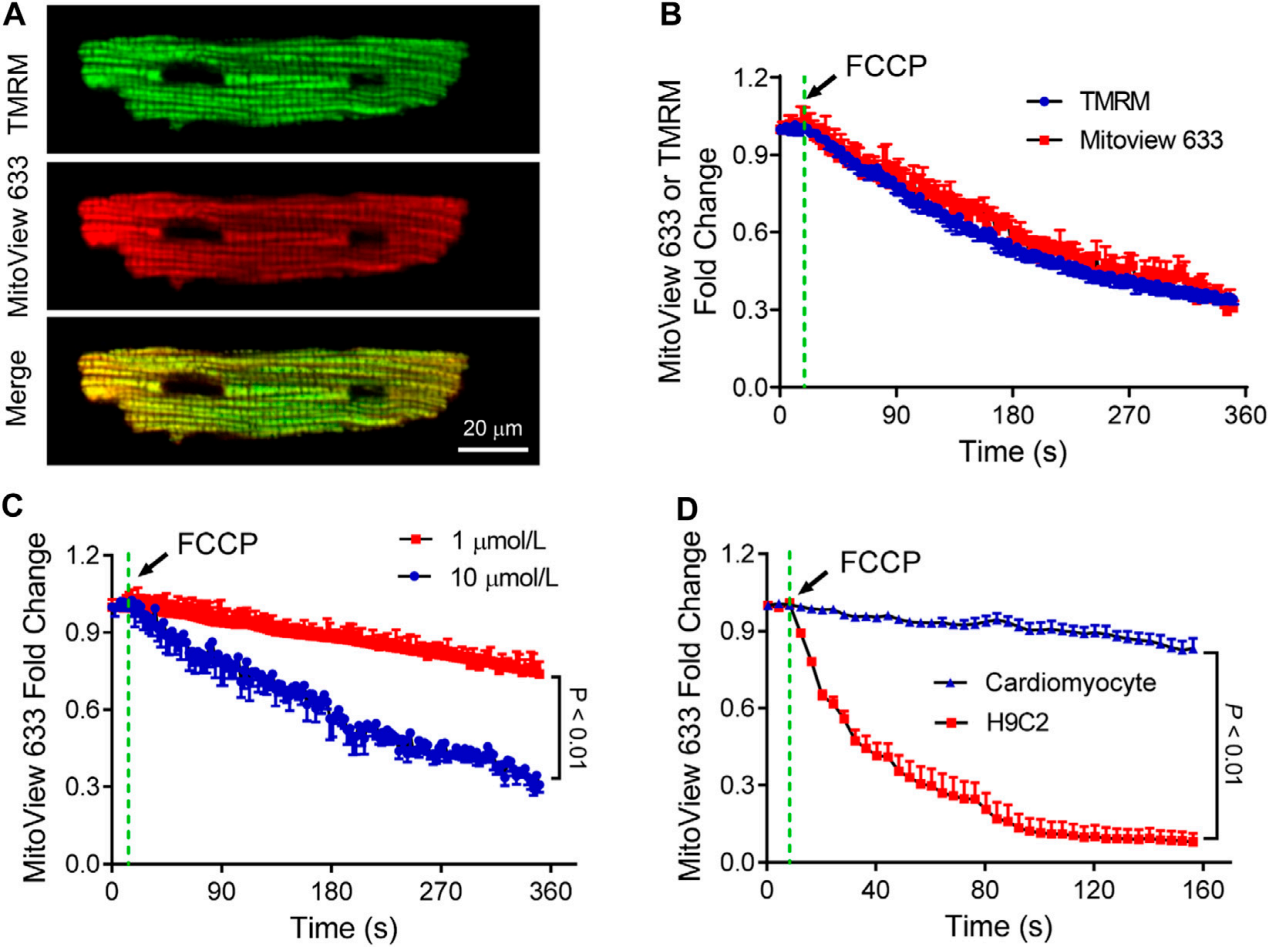
Characterization of MitoView 633 measurement of mitochondrial depolarization. **(A)** Representative confocal images showing co-staining of TMRM (Excitation: 543 nm; Emission wavelength range: 560–620 nm) and MitoView 633 (Excitation: 635 nm; Emission wavelength range: 655–675 nm) in isolated cardiomyocytes. **(B)** Comparison of FCCP perfusion (10 μmol/L)-induced cardiomyocyte mitochondrial depolarization detected by MitoView 633 (red line) and TMRM (blue line). **(C)** FCCP dose-dependent induction of mitochondrial depolarization in adult cardiomyocytes detected by MitoView 633. **(D)** Comparison of FCCP (1 μmol/L)-induced mitochondrial depolarization in H9C2 cells and adult cardiomyocytes detected by MitoView 633. Each group includes 4-6 cells from 4 mice/cell cultures.

FCCP is a potent uncoupler of mitochondrial oxidative phosphorylation, which disrupts ATP synthesis by transporting protons across the mitochondrial inner membrane, interfering with the proton gradient (Perry et al., 2011). Our subsequent investigation focused on assessing how MitoView 633 responds to FCCP treatment and comparing its dynamics with that of TMRM. To accomplish this, isolated adult cardiomyocytes were co-stained with both TMRM and MitoView 633 (Figure 4A) and subjected to FCCP (10 μmol/L) perfusion.

Simultaneous confocal imaging of TMRM and MitoView 633 revealed a similar decline in their fluorescence following FCCP treatment, with a reduction of approximately 70% occurring over a span of about 6 min (Figure 4B). In a separate experiment, cardiomyocytes loaded solely with MitoView 633 were perfused with varying concentrations of FCCP. The confocal recordings demonstrated that while MitoView 633 fluorescence intensity dropped by 70% with 10 μmol/L FCCP, consistent with data shown in Figure 3B, its fluorescence diminished only 25% when the FCCP concentration was reduced to 1 μmol/L (Figure 4C). The concentration-dependent responses of FCCP on cardiomyocyte ΔΨ_m_ measured *via* MitoView 633 are consistent with those measured by TMRM (Zhao et al., 2013). Interestingly, our data unveiled a cell-type-dependent effect of FCCP. As illustrated in Figure 4D, mitochondria in H9C2 cells depolarized significantly faster (time constant: 40 s vs. 19,291 s) and to a greater extent (93% vs. 16%) when treated with the same concentration of FCCP (1 μmol/L) compared to adult cardiomyocyte mitochondria. The cell type-dependent effect of FCCP is supported by the studies by Johnson-Cadwell et al. showing that FCCP caused rapid and substantial mitochondrial depolarization in cultured cerebellar granule neurons at a concentration as low as 0.25 μmol/L (Johnson-Cadwell et al., 2007). Altogether, our data indicate that MitoView 633 can be used as a reliable ΔΨ_m_ indicator for measuring stress-induced mitochondrial depolarization.

### Concurrent recording of MitoView 633 with other fluorescent dyes in live cell imaging

**FIGURE 5:**
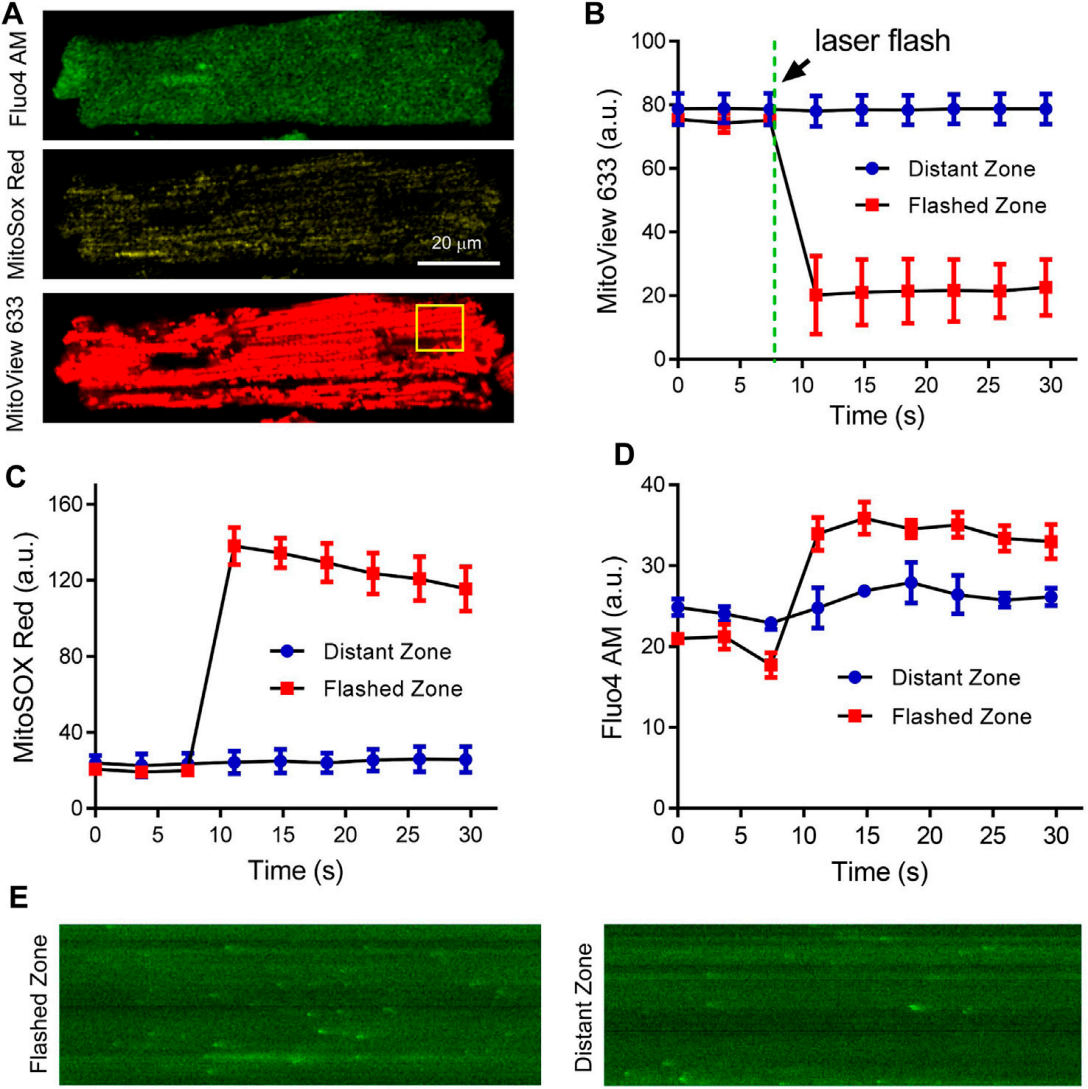
Concurrent recording of MitoView 633, MitoSox Red, and Fluo4 AM in adult cardiomyocytes. **(A)** Representative confocal images showing co-staining of Fluo4-AM (Ex/Em: 488 nm/505 nm), MitoSOX Red (Ex/Em: 515 nm/580 nm), and MitoView 633 (Ex/Em: 635 nm/664 nm) in adult cardiomyocytes. **(B, D)**: Concurrent recording of MitoView 633, MitoSOX Red, and Fluo4-AM revealed mitochondrial depolarization **(B)**, increased mitochondrial ROS **(C)**, and elevated cytosolic Ca^2+^ **(D)** within the laser flash zone (indicated by the yellow square in A) compared to the distant zone. **(E)**: Representative linescan imaging of Ca^2+^ sparks in the laser flashed zone and distant zone. 10 cells from three mice were examined.

To explore the compatibility of MitoView with other fluorescent dyes of interest, adult cardiomyocytes were co-stained with Fluo4-AM, MitoSOX Red, and MitoView 633 (Figure 5A), and continuously perfused with Tyrode’s solution. A selected cell was subjected to local laser flash (indicated by the yellow square in Figure 5A) to induce regional oxidative stress, as previously described (Goh et al., 2016), and was subsequently imaged over time. The analysis of the data revealed that ΔΨ_m_ within the flash zone experienced a rapid and substantial depolarization, while in the distant zone, it remained nearly unchanged (Figure 5B). Accordingly, mitochondrial ROS levels significantly increased in the laser-flashed zone, as evidenced by the MitoSOX Red fluorescence (Figure 5B). Notably, the MitoSOX Red fluorescent intensity exhibited a gradual decrease after the initial burst (Figure 5C), likely attributed to substantial disruption of the electron transport chain. Fluo4-AM imaging depicted an elevation in local Ca^2+^ within the flashed zone (Figure 5D), which could be attributed to mitochondrial depolarization inhibiting mitochondrial Ca^2+^ uptake and buffering. The change in Fluo4-AM fluorescent intensity in the distant zone was less pronounced. This increase in Fluo4-AM fluorescence upon laser flash might also be linked to the elevation of ROS levels. As shown in Figure 5E, Ca^2+^ spark frequency in the flashed zone was significantly higher compared to that in the distant zone, confirming increased Ca^2+^ release. This effect aligns with previous reports on the stimulatory impact of mitochondrial-derived ROS on sarcoplasmic reticulum Ca^2+^ release and spark (Yan et al., 2008; Zhou et al., 2011). The ability of MitoView 633 to be used in conjunction with MitoSOX Red could prove invaluable for laboratories employing conventional filter-based fluorescent microscopes, as the spectral characteristics of TMRM largely overlap with those of MitoSOX Red. It's worth noting that while the local flashes triggered regional mitochondrial depolarization in cardiomyocytes in our study, other reports have indicated that this approach can induce cell-wide (Papanicolaou et al., 2011), inter-cell mitochondrial depolarization (Slodzinski et al., 2008), or even sustained mitochondrial oscillations (Aon et al., 2003; Zhou et al., 2011; Goh et al., 2016), suggesting that this phenomenon is species-specific and cell status-dependent.

## Conclusion

In conclusion, this study demonstrates the utility of MitoView 633 as a reliable mitochondrial membrane potential probe, underscored by its robust performance across a practical range of experimental conditions. Its distinctive absorption and emission spectra render it compatible with various other red-spectrum dyes, enabling comprehensive investigations into mitochondrial bioenergetics and associated processes through the use of fluorescent markers. Moreover, its far-red fluorescent spectrum presents the advantage of facilitating observations at the tissue level. This characteristic allows for enhanced tissue penetration and reduced scattering compared to shorter-wavelength counterparts, making it a valuable tool for studying mitochondrial dynamics in deeper tissue layers. Additionally, it's noteworthy that while our study involved medium replacement after dye loading, MitoView 633 can also be effectively imaged without the need for a washing step, further simplifying experimental procedures. It is worth noting that like other lipophilic probes, it is possible that MitoView 633 can bind to mitochondria, causing an apparent deviation of the ΔΨ_m_ -dependent accumulation from that predicted by the Nernst equation. Future studies are warranted to address this issue and better establish MitoView 633 as a reliable tool for ΔΨ_m_ measurement.

## Data Availability

The original contributions presented in the study are included in the article/Supplementary Material, further inquiries can be directed to the corresponding author.

## References

[B1] Andersson D. C. Fauconnier J. Yamada T. Lacampagne A. Zhang S. J. Katz A. (2011). Mitochondrial production of reactive oxygen species contributes to the beta-adrenergic stimulation of mouse cardiomycytes. J. Physiol. 589, 1791–1801. 10.1113/jphysiol.2010.202838 21486840 PMC3099030

[B2] Aon M. A. Cortassa S. Marban E. O'Rourke B. (2003). Synchronized whole cell oscillations in mitochondrial metabolism triggered by a local release of reactive oxygen species in cardiac myocytes. J. Biol. Chem. 278, 44735–44744. 10.1074/jbc.M302673200 12930841

[B3] Bernas T. Zarȩbski M. Cook R. R. Dobrucki J. W. Cook P. R. (2004). Minimizing photobleaching during confocal microscopy of fluorescent probes bound to chromatin: role of anoxia and photon flux. J. Microsc. 215, 281–296. 10.1111/j.0022-2720.2004.01377.x 15312193

[B4] Bertholet A. M. Kazak L. Chouchani E. T. Bogaczynska M. G. Paranjpe I. Wainwright G. L. (2017). Mitochondrial patch clamp of beige adipocytes reveals UCP1-positive and UCP1-negative cells both exhibiting futile creatine cycling. Cell Metab. 25, 811–822. 10.1016/j.cmet.2017.03.002 28380374 PMC5448977

[B5] Contreras L. Drago I. Zampese E. Pozzan T. (2010). Mitochondria: the calcium connection. Biochimica Biophysica Acta (BBA) - Bioenergetics 1797, 607–618. 10.1016/j.bbabio.2010.05.005 20470749

[B6] Davidson M. M. Nesti C. Palenzuela L. Walker W. F. Hernandez E. Protas L. (2005). Novel cell lines derived from adult human ventricular cardiomyocytes. J. Mol. Cell Cardiol. 39, 133–147. 10.1016/j.yjmcc.2005.03.003 15913645

[B7] Ernst P. Chen K. Tang Y. Kim S. Guan J. He J. (2021). Investigation into the difference in mitochondrial-cytosolic calcium coupling between adult cardiomyocyte and hiPSC-CM using a novel multifunctional genetic probe. Pflugers Arch. 473, 447–459. 10.1007/s00424-021-02524-3 33587181 PMC8100988

[B8] Ernst P. Xu N. Qu J. Chen H. Goldberg M. S. Darley-Usmar V. (2019). Precisely control mitochondria with light to manipulate cell fate decision. Biophys. J. 117, 631–645. 10.1016/j.bpj.2019.06.038 31400914 PMC6712847

[B9] Garner D. L. Thomas C. A. (1999). Organelle-specific probe JC-1 identifies membrane potential differences in the mitochondrial function of bovine sperm. Mol. Reprod. Dev. 53, 222–229. 10.1002/(SICI)1098-2795(199906)53:2<222:AID-MRD11>3.0.CO;2-L 10331460

[B10] Goh K. Y. He L. Song J. Jinno M. Rogers A. J. Sethu P. (2019). Mitoquinone ameliorates pressure overload-induced cardiac fibrosis and left ventricular dysfunction in mice. Redox Biol. 21, 101100. 10.1016/j.redox.2019.101100 30641298 PMC6330374

[B11] Goh K. Y. Qu J. Hong H. Liu T. Dell'Italia L. J. Wu Y. (2016). Impaired mitochondrial network excitability in failing Guinea-pig cardiomyocytes. Cardiovasc Res. 109, 79–89. 10.1093/cvr/cvv230 26433944 PMC4692289

[B12] Hanninen S. L. Ronkainen J. J. Leskinen H. Tavi P. (2010). Mitochondrial uncoupling downregulates calsequestrin expression and reduces SR Ca2+ stores in cardiomyocytes. Cardiovasc Res. 88, 75–82. 10.1093/cvr/cvq180 20525644

[B13] Honda H. M. Korge P. Weiss J. N. (2005). Mitochondria and ischemia/reperfusion injury. Ann. N. Y. Acad. Sci. 1047, 248–258. 10.1196/annals.1341.022 16093501

[B14] Ishii M. Rohrer B. (2017). Bystander effects elicited by single-cell photo-oxidative blue-light stimulation in retinal pigment epithelium cell networks. Cell Death Discov. 3, 16071. 10.1038/cddiscovery.2016.71 28179989 PMC5292780

[B15] Johnson-Cadwell L. I. Jekabsons M. B. Wang A. Polster B. M. Nicholls D. G. (2007). ‘Mild Uncoupling’ does not decrease mitochondrial superoxide levels in cultured cerebellar granule neurons but decreases spare respiratory capacity and increases toxicity to glutamate and oxidative stress. J. Neurochem. 101, 1619–1631. 10.1111/j.1471-4159.2007.04516.x 17437552

[B16] Kim S. Song J. Ernst P. Latimer M. N. Ha C. M. Goh K. Y. (2020). MitoQ regulates redox-related noncoding RNAs to preserve mitochondrial network integrity in pressure-overload heart failure. Am. J. Physiol. Heart Circ. Physiol. 318, H682–H695. 10.1152/ajpheart.00617.2019 32004065 PMC7099446

[B17] Korchak H. M. Rich A. M. Wilkenfeld C. Rutherford L. E. Weissmann G. (1982). A carbocyanine dye, DiOC6(3), acts as a mitochondrial probe in human neutrophils. Biochem. Biophys. Res. Commun. 108, 1495–1501. 10.1016/s0006-291x(82)80076-4 7181903

[B18] Li Y. Zhang L. Qu T. Tang X. Li L. Zhang G. (2017). Conservation and divergence of mitochondrial apoptosis pathway in the Pacific oyster, *Crassostrea gigas* . Cell Death Dis. 8, e2915. 10.1038/cddis.2017.307 28682310 PMC5550854

[B19] Maioral M. F. Bodack C. D. N. Stefanes N. M. Bigolin Á. Mascarello A. Chiaradia-Delatorre L. D. (2017). Cytotoxic effect of a novel naphthylchalcone against multiple cancer cells focusing on hematologic malignancies. Biochimie 140, 48–57. 10.1016/j.biochi.2017.06.004 28610775

[B20] Min Y.-L. Jaichander P. Sanchez-Ortiz E. Bezprozvannaya S. Malladi V. S. Cui M. (2018). Identification of a multipotent Twist2-expressing cell population in the adult heart. Proc. Natl. Acad. Sci. 115, E8430–E8439. 10.1073/pnas.1800526115 30127033 PMC6130356

[B21] Papanicolaou K. N. Khairallah R. J. Ngoh G. A. Chikando A. Luptak I. O'Shea K. M. (2011). Mitofusin-2 maintains mitochondrial structure and contributes to stress-induced permeability transition in cardiac myocytes. Mol. Cell. Biol. 31, 1309–1328. 10.1128/MCB.00911-10 21245373 PMC3067905

[B22] Perry S. W. Norman J. P. Barbieri J. Brown E. B. Gelbard H. A. (2011). Mitochondrial membrane potential probes and the proton gradient: a practical usage guide. Biotechniques 50, 98–115. 10.2144/000113610 21486251 PMC3115691

[B23] Polster B. M. Nicholls D. G. Ge S. X. Roelofs B. A. (2014). Use of potentiometric fluorophores in the measurement of mitochondrial reactive oxygen species. Methods Enzymol. 547, 225–250. 10.1016/B978-0-12-801415-8.00013-8 25416361 PMC4484872

[B24] Reers M. Smith T. W. Chen L. B. (1991). J-aggregate formation of a carbocyanine as a quantitative fluorescent indicator of membrane potential. Biochemistry 30, 4480–4486. 10.1021/bi00232a015 2021638

[B25] Scaduto R. C. Grotyohann L. W. (1999). Measurement of mitochondrial membrane potential using fluorescent rhodamine derivatives. Biophys. J. 76, 469–477. 10.1016/S0006-3495(99)77214-0 9876159 PMC1302536

[B26] Sciuto K. J. Deng S. W. Moreno A. Zaitsev A. V. (2019). Chronology of critical events in neonatal rat ventricular myocytes occurring during reperfusion after simulated ischemia. PLoS One 14, e0212076. 10.1371/journal.pone.0212076 30730997 PMC6366697

[B27] Segev A. Garcia-Oscos F. Kourrich S. (2016). Whole-cell patch-clamp recordings in brain slices. J. Vis. Exp., 54024. 10.3791/54024 27341060 PMC4927800

[B28] Shapiro H. M. (2000). Membrane potential estimation by flow cytometry. Methods 21, 271–279. 10.1006/meth.2000.1007 10873481

[B29] Slodzinski M. K. Aon M. A. O'Rourke B. (2008). Glutathione oxidation as a trigger of mitochondrial depolarization and oscillation in intact hearts. J. Mol. Cell Cardiol. 45, 650–660. 10.1016/j.yjmcc.2008.07.017 18760283 PMC2604133

[B30] Sorgato M. C. Keller B. U. Stuhmer W. (1987). Patch-clamping of the inner mitochondrial membrane reveals a voltage-dependent ion channel. Nature 330, 498–500. 10.1038/330498a0 2446143

[B31] Uttara B. Singh A. V. Zamboni P. Mahajan R. T. (2009). Oxidative stress and neurodegenerative diseases: a review of upstream and downstream antioxidant therapeutic options. Curr. Neuropharmacol. 7, 65–74. 10.2174/157015909787602823 19721819 PMC2724665

[B32] Wallace D. C. (2012). Mitochondria and cancer. Nat. Rev. Cancer 12, 685–698. 10.1038/nrc3365 23001348 PMC4371788

[B33] Wei A. C. Liu T. Cortassa S. Winslow R. L. O'Rourke B. (2011). Mitochondrial Ca2+ influx and efflux rates in Guinea pig cardiac mitochondria: low and high affinity effects of cyclosporine A. Biochim. Biophys. Acta 1813, 1373–1381. 10.1016/j.bbamcr.2011.02.012 21362444 PMC3109245

[B34] Xu W. Liu Y. Wang S. McDonald T. Van Eyk J. E. Sidor A. (2002). Cytoprotective role of Ca2+- activated K+ channels in the cardiac inner mitochondrial membrane. Science 298, 1029–1033. 10.1126/science.1074360 12411707

[B35] Yan Y. Liu J. Wei C. Li K. Xie W. Wang Y. (2008). Bidirectional regulation of Ca2+ sparks by mitochondria-derived reactive oxygen species in cardiac myocytes. Cardiovasc Res. 77, 432–441. 10.1093/cvr/cvm047 18006452

[B36] Zhao M. Fan C. Ernst P. J. Tang Y. Zhu H. Mattapally S. (2018). Y-27632 preconditioning enhances transplantation of human-induced pluripotent stem cell-derived cardiomyocytes in myocardial infarction mice. Cardiovasc. Res. 115, 343–356. 10.1093/cvr/cvy207 PMC6341224 30107391

[B37] Zhao Z. Gordan R. Wen H. Fefelova N. Zang W. J. Xie L. H. (2013). Modulation of intracellular calcium waves and triggered activities by mitochondrial ca flux in mouse cardiomyocytes. PLoS One 8, e80574. 10.1371/journal.pone.0080574 24348912 PMC3857829

[B38] Zhou L. Aon M. A. Almas T. Cortassa S. Winslow R. L. O'Rourke B. (2010). A reaction-diffusion model of ROS-induced ROS release in a mitochondrial network. PLoS Comput. Biol. 6, e1000657. 10.1371/journal.pcbi.1000657 20126535 PMC2813265

[B39] Zhou L. Aon M. A. Liu T. O'Rourke B. (2011). Dynamic modulation of Ca(2+) sparks by mitochondrial oscillations in isolated Guinea pig cardiomyocytes under oxidative stress. J. Mol. Cell Cardiol. 51, 632–639. 10.1016/j.yjmcc.2011.05.007 21645518 PMC3179563

[B40] Zhou L. Cabrera M. E. Huang H. Yuan C. L. Monika D. K. Sharma N. (2007). Parallel activation of mitochondrial oxidative metabolism with increased cardiac energy expenditure is not dependent on fatty acid oxidation in pigs. J. Physiol. 579, 811–821. 10.1113/jphysiol.2006.123828 17185335 PMC2151353

[B41] Zhou L. O'Rourke B. (2012). Cardiac mitochondrial network excitability: insights from computational analysis. Am. J. Physiol. Heart Circ. Physiol. 302, H2178–H2189. 10.1152/ajpheart.01073.2011 22427517 PMC3378299

